# Roles of Exosomes in Chronic Rhinosinusitis: A Systematic Review

**DOI:** 10.3390/ijms231911284

**Published:** 2022-09-25

**Authors:** Karolina Dżaman, Katarzyna Czerwaty

**Affiliations:** Department of Otolaryngology, Centre of Postgraduate Medical Education, Marymoncka 99/103, 01-813 Warsaw, Poland

**Keywords:** exosomes, extracellular vesicles, exosomal biomarkers, chronic rhinosinusitis, microRNA, liquid biopsy, biomarker, sinusitis

## Abstract

The pathophysiology of chronic rhinosinusitis (CRS) is multifactorial and not entirely clear. The objective of the review was to examine the current state of knowledge concerning the role of exosomes in CRS. For this systematic review, we searched PubMed/MEDLINE, Scopus, CENTRAL, and Web of Science databases for studies published until 7 August 2022. Only original research articles describing studies published in English were included. Reviews, book chapters, case studies, conference papers, and opinions were excluded. The quality of the evidence was assessed with the modified Office and Health Assessment and Translation (OHAT) Risk of Bias Rating Tool for Human and Animal Studies. Of 250 records identified, 17 were eligible, all of which had a low to moderate risk of overall bias. Presented findings indicate that exosomal biomarkers, including proteins and microRNA, act as promising biomarkers in the diagnostics and prognosis of CRS patients and, in addition, may contribute to finding novel therapeutic targets. Exosomes reflecting tissue proteomes are excellent, highly available material for studying proteomic alterations noninvasively. The first steps have already been taken, but more advanced research on nasal exosomes is needed, which might open a wider door for individualized medicine in CRS.

## 1. Introduction

Chronic rhinosinusitis (CRS) is defined as persistent inflammation and can be differentiated clinically into CRSwNP and CRSsNP, but currently, a classification based on endotypes is in use, better reflecting pathomechanisms involved in this heterogeneous disease, which allows more personalized treatment [1,2,3].

Extracellular vesicles (EVs) are membranous vesicles of endocytic origin that can be released from almost all host cell types and cannot replicate (do not contain a functional nucleus) [4]. Depending on their intracellular origin, EVs can be classified into exosomes, microparticles, and apoptotic bodies. Exosomes are small intraluminal vesicles (30–150 nm) formed by the inward budding of the endosomal membrane during the maturation of multivesicular bodies (MVBs) and are released into the extracellular space and body fluids as a result of the fusion of MVBs with the cell membrane [5]. Exosomes carry cell-specific cargos of proteins, lipids, nucleic acids, amino acids, and metabolites, which can reflect their cell of origin and be transmitted to distant from their cells of origin and thereby participate in intercellular communication [5,6,7].

EVs are present in diverse human body fluids. Due to the nasal function of filtering the inhaled air, nasal exosomes are the first line of defense against inhaled particles, such as air pollutants or allergens, and knowing their biological functions can be crucial for better understanding the pathophysiology of CRS. It seems that molecular components of exosomes in the nasal cavity are altered in CRS [8]. Exosomes can be isolated using ultracentrifugation (UC) and purification [9,10]. Recently, exosomes are seen as therapeutic targets and potential biomarkers in cancers [6], but their role in inflammatory diseases is less known. The discovery of the exchange of cellular components through exosomes allows for further studies in designing exosome-based therapeutics [7]. 

This article presents an up-to-date review of studies that were conducted to evaluate the role of exosomes in CRS. This is the first systematic review aiming to determine the exosomal biomarkers researched in CRS. The obtained data will facilitate an improved understanding of the potential of exosomal biomarkers in diagnostics and the treatment of CRS patients. 

## 2. Methods

### 2.1. Methods Literature Retrieval

**Figure 1:**
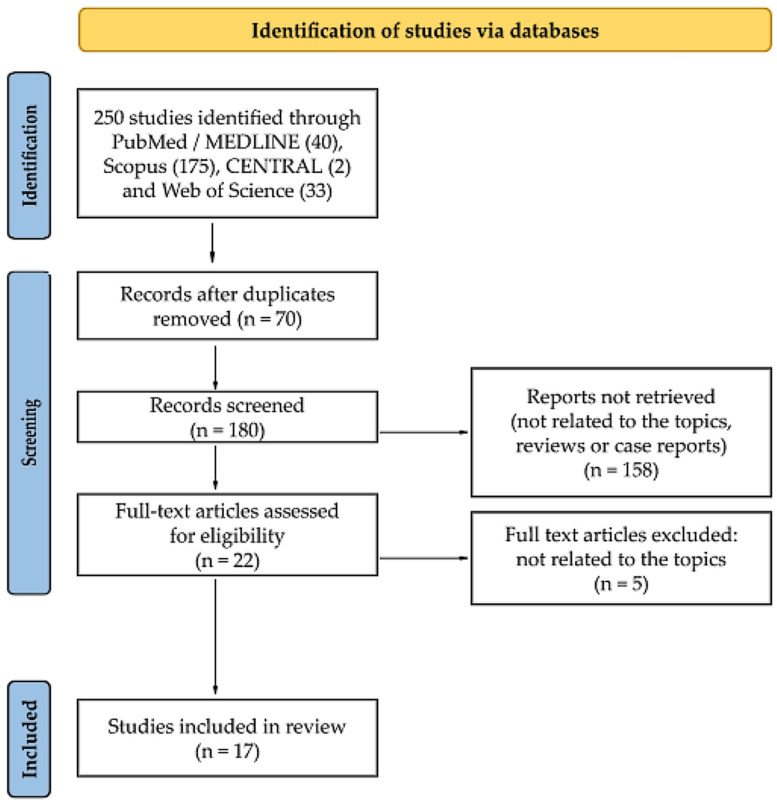
Flow diagram of the systematic literature search. The Preferred Reporting Items for Systematic Reviews and Meta-Analyses (PRISMA) flow diagram shows the study selection process.

This study was conducted by the Preferred Reporting Items for Systematic Reviews and Meta-Analyses (PRISMA) statement published in 2020 [11]. A flow chart is provided in Figure 1. A literature search for this review was performed using the following keywords: “exosome”, “exosomes”, “extracellular vesicle”, “extracellular vesicles”, “exosomal”, “liquid biopsy”, and “sinusitis” or “rhinosinusitis”. Four electronic databases were searched: MEDLINE (through PubMed), Scopus, CENTRAL, and Web of Science. The search strategy for each database is presented in Table S1. The last search was performed on 7 August 2022 on each database. The authors also carried out a “snowball” search to identify additional studies by searching the reference lists of publications eligible for full-text review; however, no additional records meeting the inclusion criteria were noted. It was not necessary to contact the authors of the retrieved research articles for additional information. The methods of the analysis and inclusion criteria were specified in advance. The inclusion and exclusion criteria are summarized in Table 1.

Duplicates were removed using the automatic EndNote 20 duplicate finder, followed by a manual search. An eligibility assessment was performed independently in an unblinded standardized manner by two reviewers. The researchers screened the titles and abstracts of all articles retrieved. In case of disagreement, consensus on which articles to screen full-text was reached by discussion. Next, two researchers independently screened full-text articles for inclusion. Again, in case of disagreement, the consensus was reached on inclusion or exclusion by discussion. We selected papers that concerned the exosomal biomarkers in CRS. The results of relevant original studies published in the English language have been summarized and discussed in this consistent review. 

### 2.2. Data Extraction

The eligibility of all the studies was evaluated, and the data of each study were retrieved individually by two investigators, with disagreements resolved using discussion and consensus. These included the following: (1) baseline information, including the first author’s name, year of publication, and country of the study; (2) study design, including the source of exosomes, numbers of study and control groups, exosome isolation and characterization methods, exosomal markers, and main outcomes.

### 2.3. Assessment of Quality of Studies

The risk of bias was assessed in the included studies using the modified Office and Health Assessment and Translation (OHAT) Risk of Bias Rating Tool for Human and Animal Studies (https://ntp.niehs.nih.gov/ntp/ohat/pubs/riskofbiastool_508.pdf, accessed on the 15 August 2022). Five types of bias (selection, performance, attrition/exclusion, detection, and selective reporting) were rated independently by two review authors based on a four-point scale: “definitely low risk of bias”, “probably low risk of bias”, “probably high risk of bias”, and “definitely high risk of bias”. Any discrepancies in judgments regarding the risk of bias or justifications for judgments were resolved by discussion to reach a consensus between the two review authors. 

## 3. Results and Discussion

### 3.1. Search Results

Details regarding the selection process are summarized in a custom-built PRISMA flow chart in Figure 1. A systematic literature search retrieved 250 citations from PubMed, Scopus, CENTRAL, and Web of Science. Of these, 70 publications were identified as duplicates and so were eliminated. After reading the titles and abstracts, 158 records were eliminated as there was no association between exosomes and CRS, review articles, or case studies. The verification of full-texts excluded five articles as they were out of topic. As a result of the described search procedure, 17 articles that met all inclusion criteria were retrieved [8,12,13,14,15,16,17,18,19,20,21,22,23,24,25,26,27].

### 3.2. Study Characteristics and Study Quality

**Figure 2:**
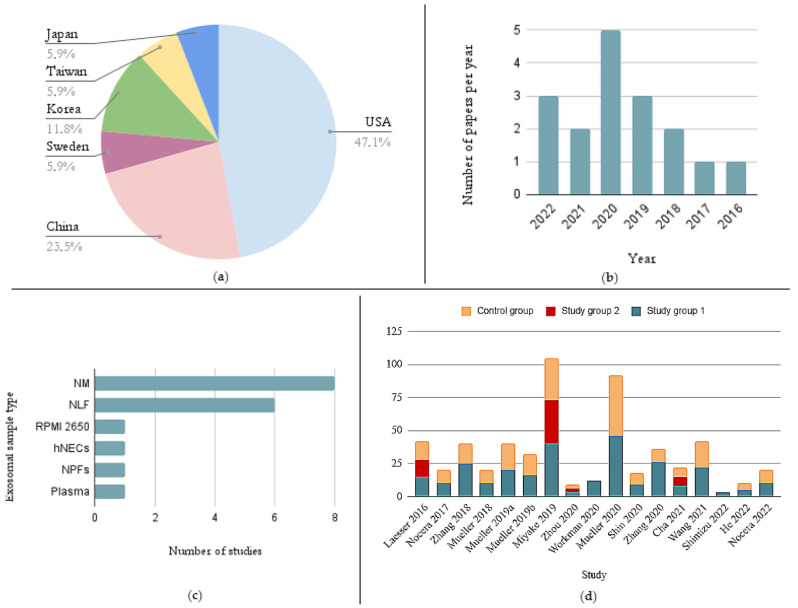
Basic information about retrieved papers. (**a**) Countries where the selected studies have been performed. (**b**) Number of papers concerning exosomes in CRS per year. (**c**) Use of particular exosomal samples in included studies. (**d**) Group sizes in the included studies [8,12,13,14,15,16,17,18,19,20,21,22,23,24,25,26,27] (2019a [15], 2019b [16]).

Basic data on the research works included in this systematic review are collected in Figure 2 and Table 2. All of the articles finally selected for the review were original studies published in English. Eight studies were conducted in the USA, four in China, two in Korea, and one in Sweden, Taiwan, and Japan (Figure 2a). All 17 retrieved papers were published in the years 2016–2022, and most of them were published in in 2020 (Figure 2b). 

The most frequently used exosomal source was nasal mucus (NM) (in eight studies), followed by nasal lavage fluid (NLF) (in six studies). Other exosomal samples such as plasma, nasal polyp fibroblasts (NPFs), primary human nasal epithelial cells (hNECs), and human nasal epithelial cell line RPMI 2650 (American Type Culture Collection CCL-30) were used once (Figure 2c). The sample size ranged from 3 to 105 (Figure 2d, Table 2). Sixteen studies included clinical sample-derived exosome components in their design, and the only exception was Shin’s study [22]. Fourteen studies compared exosomes from clinical cohorts [8,12,13,14,15,16,17,18,19,20,23,25,26,27]. In seven studies primary or established cell lines were used [21,22,25,26,27,28,29]. We have included these studies, as they may be considered models of a situation occurring in the case of CRS. The main inclusion criteria entailed studies describing the role of exosomes in CRS, although we excluded studies concerning EVs derived from bacteria instead of human cells. 

Due to the variety of the included study types, the risk of bias in the individual studies was assessed using the above-described OHAT Tool adopted by the authors for the needs of this review (https://ntp.niehs.nih.gov/ntp/ohat/pubs/riskofbiastool_508.pdf, accessed on 15 August 2022). The evaluation results are summarized in Supplementary Table S2. All retrieved studies had a low to moderate risk of overall bias.

### 3.3. Method of Mucus and NLF Collection

Mucus samples were taken before antibiotic/steroid administration or tissue sampling by applying compressed polyvinyl alcohol sponges into nasal cavities, most frequently against the middle meatus and adjacent to the middle turbinate for five minutes, while taking care to not abrade the mucosa or contaminate the sponges with blood [14,15,16,17,18,19,20,24].

NLF was mainly collected using established methods with minor adjustments [10,30]. In Wang’s [23] and Cha’s [12] studies, NLFs were taken immediately before operation when the patients were under general anesthesia with oral intubation. A warm saline solution was instilled and irrigated into the nostrils with a syringe. Subsequently, NLFs were gathered by aspirating the lavage fluids into another empty syringe or in a screw bottle. Bleeding of mucosa was avoided during the aspiratin [12,23]. In Cha’s study additionally, a three-way catheter was inserted into the nasal cavity to the end for nasal irrigation, ballooned in the posterior choana area, pulled forward so that the irrigation saline did not fall down the neck, and then ballooned in the nostril area, and irrigation was repeated until the total irrigation sample was at least 10 mL [12]. In other studies, NLF collection was performed without anesthesia. Mostly, 0,9% saline was instilled in the nostril of the person while the head leaned back at an angle of about 30° and the soft palate closed. The NLF was accumulated by passive dripping of the fluid into a container when subjects tilted their head forward [8,25,26,27].

### 3.4. Overview of EV Isolation Methodologies and Characterisation

Various EV isolation and purification techniques were reported with UC being the most common method for EV isolation and immunoblotting and transmission electron microscopy (TEM) for the characterization and classification of EVs (Table 3).

The choice of a proper isolation method has a profound impact on the identification of exosomal-specific functions and biomarkers [31]. Among EV separation methods UC, density gradient centrifugation, size-exclusion chromatography, and separation using polymers can be distinguished [12]. UC is a method in which a very high speed is used to generate centrifugal force as high as 100,000× *g* [31]. It has been proven that ultracentrifugation is a superior method for the isolation of EVs compared to a commercially available precipitation method (ExoQuick, System Biosciences, Palo Alto, CA, USA) because provides greater purity with higher protein and exosome yield [19,31]. Individual researchers used various centrifugation parameters such as centrifugal acceleration and time or filter sizes. In Table 3, specific information about used in particular studies methods can be found. Individual protocols differ in the speed of the separation process and, sometimes, the number of centrifugation steps. In all included studies that investigated NM samples [14,15,16,17,18,19,20,24], UC was conducted following the protocol proposed by Thery [9]. This method allows successive separations: firstly, cells, then dead cells, debris, and at the end, contaminating proteins for finally isolating exosomes.

The advantages of UC are reduced contamination risks, large sample capacity, yielding large amounts of exosomes, but disadvantages include high equipment cost, long run times, and labor intensiveness. There is also a risk that high-speed centrifugation can damage exosomes [31,32]. Ultrafiltration is a fast method that does not require special equipment, but on the other hand, it provides moderate purity in terms of the isolated exosomes, shear-stress-induced deterioration, and exosome loss because of its attachment to membranes [31]. Size exclusion chromatography allows for the precise separation of large and small molecules, without affecting exosome structure. Unfortunately, this method requires a longer time periods [31].

**Table 3:** Overview of purification, characterization, and exosomal markers used in included studies.

Study	Isolation Method	Exosome Characterization	Exosome Markers	
			Positive	Negative
**Lasser (2016)** [8]	UC described by Lasser [10] 1. Centrifugation at 300× *g* for 10 min at 4 °C. 2. Storage at −80 °C. 3. Thawing and transferring to ultracentrifuge tubes filled with PBS. 4. Centrifugation at 16,500× *g* for 20 min at 4 °C. 5. Filtration 0.2-µM filter. 6. Ultracentrifugation at 120,000× *g* for 70 min at 4 °C.	FCM, WB	CD9, CD14, CD63, TSG101, NOS2, S100A8	Calnexin
**Nocera (2017)** [19]	UC described by Thery [9] 1. Centrifugation at 1500× *g* for 30 min at 4 °C. 2. Dilution in 150 µL of PBS with Protease Inhibitor Cocktail. 3. Centrifugation at 12,000× *g* for 45 min at 4 °C. 4. Suspending in 4.5 mL of PBS in polypropylene tubes. 5. Ultracentrifugation at 110,000× *g* for 2 h at 4 °C. 6. Resuspend in 4.5 mL of PBS. 7. Filtration 0.22-µM filter. 8. Centrifugation at 110,000× *g* for 70 min at 4 °C.	ELISA, TEM	CD9, CD63	NR
**Zhang (2018)** [25]	UC described by Valadi [33] and Romancino [34] 1. Centrifugation at 6000× *g* for 30 min at 4 °C. 2. Centrifugation at 10,000× *g* for 60 min at 4 °C. 3. Filtration 0.2-µM filter and qEV size-exclusion columns. 4. Ultracentrifugation at 100,000× *g* for 60 min at 4 °C.	TEM, NTA, WB	CD9, CD63, β-actin	NR
**Mueller (2018)** [17]	UC described by Thery [9] (see above)	SOMAscan	NR	NR
**Mueller (2019)** [15]	UC described by Thery [9] (see above)	WB, SOMAscan	GAPDH	NR
**Mueller (2019)** [16]	UC described by Thery [9] (see above)	WB	GAPDH	NR
**Miyake (2019)** [14]	UC described by Thery [9] (see above)	ELISA	NR	NR
**Zhou (2020)** [27]	1. Centrifugation at 1000× *g* for 10 min. 2. Centrifugation at 16,500× *g* for 30 min at 4 °C. 3. Ultracentrifugation at 100,000× *g* for 2 h at 4 °C. 4. Resuspend in 4.5 mL of PBS. 5. Filtration 0.2-µM filter.	NTA, TEM, WB	CD9, TSG101, GAPDH	NR
**Workman (2020)** [24]	UC described by Thery [9] (see above)	SOMAscan	NR	NR
**Mueller (2020)** [18]	UC described by Thery [9] (see above)	SOMAscan	NR	NR
**Shin (2020)** [22]	1. Centrifugation at 200× *g* for 10 min at 4 °C. 2. Centrifugation at 2000× *g* for 20 min at 4 °C. 3. Centrifugation at 10,000× *g* for 30 min at 4 °C. 4. Filtration 0.22-µM filter 5. Centrifugation at 100,000× *g* for 70 min. 6. Washing twice in PBS by centrifugation at 100,000× *g* for 70 min.	NR	NR	NR
**Zhang (2020)** [26]	UC described by Valadi [33] and Romancino [34] (see above)	WB, TEM, NTA	CD9, CD63, ALIX, TSG101	GM130
**Cha (2021)** [12]	1. Centrifugation at 300× *g* for 10 min at 4 °C. 2. Centrifugation at 2000× *g* for 20 min. 3. Centrifugation at 16,500× *g* for 50 min. 4. Centrifugation at 120,000× *g* for 60 min. 5. Vortexing with 1 mL of ice-cold PBS.	FCM, TEM	CD9, CD63	NR
**Wang (2021)** [23]	UC described by Lasser [10] with minor modification (see above)	NTA, WB, TEM	CD9, CD63, Annexin V, TSG101	NR
**Shimizu (2022)** [21]	1. Centrifugation at 1500× *g* for 5 min. 2. Centrifugation at 14,000× *g* for 5 min. 3. Centrifugation at 100,000× *g* for 60 min.	ELISA, NTA, TEM	CD9, CD63	NR
**He (2022)** [13]	1. Centrifugation at 5000× *g* for 20 min below 4 °C. 2. Filtration 0.45-µM membrane. 3. Using Exosome Isolation Kit (Abace Biotechnology, Beijing, China).	NTA, WB, TEM	CD9, CD63, TSG101, ALIX	NR
**Nocera (2022)** [20]	UC described by Thery [9] (see above)	WB, SOMAscan	GAPDH	NR

ELISA—enzyme-linked immunosorbent assay; FCM—flow cytometry; GAPDH—glyceraldehyde 3-phosphate dehydrogenase; NR—not reported; NTA—nanoparticle tracking analysis; PBS—phosphate-buffered saline; UC—ultracentrifugation; WB—Western blot; TEM—transmission electron microscopy.

### 3.5. Abundance and Morphology of CRS-Derived EVs

Several EV studies suggest morphological or volume differences between EVs isolated from healthy individuals and patients with CRS. NLF flow cytometry conducted by Cha et al. demonstrated a significant increase in CD63+ or both CD9+ and CD63+ EVs in a group of CRS patients relative to the HS (healthy subjects without CRS) group, but a meaningless difference was observed between CRSsNP and CRSwNP patients [12].

Furthermore, researchers have various observations about EV concentrations in collected samples. In the study conducted by Nocera et al., there was no significant difference in median total NM-EVs concentration between CRSwNP and HS groups [19]. In contrast, Cha et al. did not notice significant differences in the NLF-EV concentration according to sex or age, but the mean exosome count per 10 mm^2^ analyzed by TEM was 75 in CRSwNP, 66 in CRSsNP, and three were in the HS group [12]. Moreover, Cha et al. found out that the expression of EV RNA, especially microRNA (miRNA), significantly increased in NLF from CRS patients [12]. In Shimizu’s study, the average count of exosomes was 6.61 × 10^8^ particles /mL, and the concentration of exosomes was 510.8 pg/mL [21].

The TEM of the isolated EVs showed variance in their size depending on the EVs source. The majority of NLF-EVs were within 30–150 nm [25,29,35]. For example, in Shimizu’s study, it was 133 nm [21]. In Wang’s study, most of the NLF-EVs of CRS patients and HS group analyzed by nanoparticle tracking analysis (NTA) had a size between 50 and 250 nm, with a similar mean diameter size for CRSsNP (204 nm) and CRSwNP (205 nm) [23]. However, in He’s study the mean diameter of plasma-derived EVs indicated by NTA was 110 nm [13]. Nocera et al. noticed NM-derived exosome size as 30–150 nm among both the control and CRSwNP [19]. Zhou et al. demonstrated the size of exosomes identified in hNECs culture medium as follows: in CRSwNP with and without coexisting asthma 92 ± 20.5 nm, in HS group 106 ± 27 nm [27]. Some authors noticed smaller sizes of NLF-derived Evs in the CRS group compared to HS and differences in size depending on CRS’s type. According to Cha’s study, the median NLF-EV size analyzed by TEM was 132 nm in the CRSwNP group, 82 nm in CRSsNP patients, and 184 nm in the HS group [12], whereas in Zhou’s study, the mean NLF-derived particle size was 316 ± 168 nm in CRSwNP patients, 312 ± 154 nm in CRSwNP with coexisting asthma, and 228 ± 119 nm in HS group [27].

Only a few authors mentioned the shape of EVs. Nocera et al. isolated spherical NM-derived exosomes [28] as well as He et al. who revealed plasma-derived exosomes membrane-bound spherical structures [17]. Zhou’s study showed exosomes derived from NLF and hNECs as circular or elliptical with a double-layered lipid molecular structure surrounding the particles [27]. Cha et al. established that NLF-EV morphology was not altered in CRS [12].

Interestingly, In Shimizu’s study, exosome secretion was stimulated by hypoxia [21]. We already know that hypoxia can change not only the secretion amount but also the size of exosomes and the expression of exosome cargos [36].

### 3.6. Differential Expression of CRS-Derived EV Surface and Cargo Proteins

In 2016, Lasser et al. provided the first description of the proteome of nasal exosomes, identified 604 proteins and analyzed them using the Gene Ontology (GO) Term Finder [8,37]. Now, we know that obtaining a detailed description of proteomes using the application of exclusion lists may be helpful.

Mueller et al. demonstrated that the overall exosomal proteome correlates more strongly between CRSwNP and HS group than with either the mucus or tissue proteome, which indicated reproducible exosomal protein expression data between patients. Additionally, the intersubject variability in proteomes was significantly lower in the exosomes than in matched whole mucus samples. It was also shown that the CRSwNP exosomal proteome overlapped with 80 tissue proteins and only 4 proteins in matched whole mucus. Mueller et al. compared the exosomal proteome in CRSwNP to the HS group and found that 75 proteins were significantly upregulated and 48 proteins were significantly downregulated [15].

Zhou et al. analyzed the differentially expressed proteins from hNECs-EVs and identified 4813 proteins, including 3262 proteins related to exosomes. Component analysis (KEGG, Kyoto Encyclopedia of Genes and Genomes) revealed proteins involved in participation in cell signal transduction (n = 479), immune system signaling (n = 256), and responses to viruses and bacteria (n = 222). They further demonstrated that in hNECs-derived exosomes from CRSwNP patients, protein changes involve mainly structural adhesion and proliferation and metabolic activity. GO pathway analysis showed increased extracellular structure organization and extracellular matrix organization in CRSwNP patients versus the HS group and KEGG analysis revealed increased p53 signaling pathway and ECM-receptor interaction in CRSwNP patients versus the HS group [27].

In another study, Mueller et al. demonstrated that pappalysin A (PAPP-A), also known as insulin-like growth factor binding protein-4 protease, is strongly upregulated in tissue and NM-derived exosomes in CRSwNP patients versus HS group, and its function was also shown to be increased in tissue as well as exosomes. The whole-transcriptomic analysis confirmed the significant upregulation of PAPP-A in CRSwNP. The transcriptomic data using qPCR revealed a significant upregulation of PAPP-A in CRSwNP; a significant downregulation with respect to inhibitor stanniocalcin-1 (STC-1), STC-2, and insulin-like growth factor binding protein–5 (IGFBP-5); and no differences for IGFBP-4 and insulin-like growth factor-1 (IGF-1) between CRSwNP and HS group. Immunohistochemistry (IHC) showed that PAPP-A is localized to the apical epithelium and in the glands of the lamina propria of nasal polyps, eosinophils, and mast cells. IGFBP-4, IGFBP-5, STC-1, and STC-2 revealed a colocalization relative to PAPP-A in nasal polyps [18]. Moreover, in Workman’s study, PAPP-A demonstrated the largest decrease in protein concentration after steroid exposure among 1300 proteins [24].

Upregulated tissue and exosomal PAPP-A in CRSwNP observed by Mueller may indicate the potential role in the promotion of epithelial proliferation and polyp growth [18]. PAPP-A was identified and described in 1974 as the pregnancy-associated plasma protein-A found in high concentrations in the circulation of pregnant women [38]. However, the role of PAPP-A was also confirmed in many cancers [39], pulmonary disease [40], ischemic cerebrovascular disease [41], and asthma [42]. The study suggests that PAPP-A may be a useful biomarker for predicting airway remodeling and reflecting the response to treatment [42]. There is a potential proinflammatory role of PAPP-A in connecting with IGF-I/PI3K/Akt signaling pathway in macrophage activation [43]. It is hypothesized that PAPP-A might be a potential therapeutic target for indirectly inhibiting IGF signaling in tissues [44].

Another exosomal protein content that has been measured and explored as a potential indicator of CRS severity was mucin 5AC. Wang’s study revealed that mucin 5AC, the primary gel-forming mucin in airways, was selectively upregulated in NLF-derived exosomes of CRSwNP relative to CRSsNP patients and was also more significantly expressed in tissue lysates in the CRSwNP samples compared to CRSsNP. In an IHC analysis on paraffin-embedded tissue specimens from CRSwNP patients, mucin 5AC was mostly expressed at the upper epithelial layers [23]. The mucin 5AC is significantly increased in the mucosa of CRS patients and can be regulated by epidermal growth factor (EGF), IL-19, IL-17A, IL-4, and IL-13 [45,46,47]. It is known that cigarette smoke or short-term fine particulate matter (PM) exposure can increase mucin 5AC expression [48,49].

Zhou et al. presented overlapping exosomal proteins found in both patients with CRSwNP and CRSwNP with coexisting asthma compared with the HS group. Upregulated in both groups included the following: plasminogen activator inhibitor 1, neutrophil defensin 1, G-protein coupled receptor family C group 5 member B, and tetraspanin-8. In contrast, in both CRS groups they observed downregulation in vacuolar protein sorting-associated protein 35, guanylate-binding protein 6, phospholipid transfer protein, and p53 apoptosis effector related to PMP-22 [27].

### 3.7. Directed MicroRNA Cargo of CRS-Derived EVs

MiRNAs are a class of small noncoding RNA molecules and posttranscriptional regulators of gene expression in physiological and pathological processes, which bind to complementary target mRNAs. In 2011, for the first time, Lasser et al. proved that NLF contains exosomes, and miRNAs are cargo molecules in these exosomes [10]. Exosomal miRNA can be transported to other cells and can be functional in this new location [33]. Plasma-derived exosomes also contain miRNA, which can be transported in plasma and mediate cell-to-cell communication [7]. EVs make it possible for cells to exchange miRNA, which may have a regulative impact on innate and adaptive immune cells [50].

Cha et al. observed that the amount of miRNA significantly increased in NLF from CRS patients relative to HS [12]. The authors presented the miRNA expression of 798 miRNAs from the NLF-derived EV in CRSwNP, CRSsNP, and HS groups. It was noticed that twelve miRNAs were differentially expressed in exosomes from CRS patients relative to HS group, including seven upregulated miRNAs (miR-15a-5p, miR-671-3p, miR-142-3p, miR-25-3p, miR-223-3p, miR-23a-3p, and miR-941) and five downregulated miRNAs (miR-1285-3p, miR-1469, miR-450a-1-3p, miR-650, and miR-664b-5p). Furthermore, eight miRNAs (miR-890, miR-519a-5p, miR-1254, miR-548t-3p, miR-1290, miR-548l, miR-376c-5p, and miR-548q) were differentially expressed in the NLF-derived exosomes of CRSwNP relative to CRSsNP patients. The differences in miRNA expression observed among phenotype groups suggest that the miRNA is relevant in CRS and may contribute considerably to the CRS’s inflammatory profile [12]. Xuan et al. conducted a study to compare the miRNA expression profiles in the SM of CRSwNP patients and HS and identified 5 upregulated (miR-210-5p, miR-3178, miR-585-3p, miR-3146, and miR-320e) and 19 downregulated miRNAs (miR-32-3p, miR-1299, miR-3196, miR-3924, miR-548e-3p, miR-3184-5p, miR-375, miR-23a-5p, miR-377-5p, miR-574-5p, miR-3149, miR-500a-5p, miR-125b-2-3p, miR-1914-5p, miR-532-3p, miR-612, miR-1298-5p, miR-1226-3p, and miR-668-3p) in CRSwNP versus HS [51]. In a similar study comparing the miRNA content of EVs from the nasal tissue of CRS patients and HS, Xia et al. showed that in all CRS patients, miR-125b, miR-155, and miR-146 were upregulated, while miR-92a, miR-26b, and miR-181b were downregulated versus HS group. Further evidence of miRNA delivery by exosomes showed that miR-125b and miR-155 were significantly upregulated in CRSwNP relative to CRSsNP [52]. Xuan’s and Xia’s studies produced divergent results, which may be incomprehensible and need further studies. Zhang et al. compared miRNA’s expression in SM from CRSsNP, eosinophilic CRSwNP (ECRSwNP), and HS using miRNA microarrays and found that miRNA-125b was upregulated in ECRSwNP [53], which is consistent with Xia et al.’s [52] reports. Korde et al. found an inverse correlation between serum mi-R-1 levels and SM eosinophilia in CRS patients, which could be crucial from a diagnostic point of view [54]. Interestingly, some studies found that NM-derived EV miRNA expression is altered in AR as well, which can be relevant in the development of AR and useful as biomarkers in patients with AR or asthma [55,56,57,58].

Apart from NM and NLF studies, miRNA profiling in CRS was performed in plasma-derived exosomes as well. He et al. [13] identified 1692 known miRNAs and 1068 novel miRNAs in plasma-derived exosomes and determined 159 significantly dysregulated miRNA transcripts, including 93 upregulated and 66 downregulated transcripts that were differentially expressed in CRSwNP relative to the HS group. The top three upregulated miRNAs were novel_miR_677, novel_miR_1037, and novel_miR_79, whereas the top three downregulated miRNAs were novel_miR_192, novel_miR_1022, and novel_miR_4 [13].

Moreover, the researchers analyzed the EVs miRNA target and its effect on biological pathways. Cha et al. [12] revealed some biological pathways enriched by upregulated genes in NLF-EVs of CRSwNP involved in CRSwNP development: Hippo signaling pathway, TGF-β signaling pathway, FoxO signaling pathway PI3K-Akt signaling pathway, Mucin-type O-glycan biosynthesis, adherens junction, and Rap1 signaling pathway.

Similarly, the O-glycan biosynthesis pathway was significantly enriched in CRSwNP in Xuan’s study [51]. O-glycan contributes to the protective functions of mucins [59], which are components in mucus secretions covering the surface of SM. On the other hand, in contrast to Cha’s study, Xuan et al.’s miRNA profile analysis demonstrated that the TGF-β signaling pathway was significantly linked to downregulated miRNAs [51]. TGF-β is a crucial immunoregulatory cytokine, which plays a role in suppressing T cells and mediating repair responses that lead to tissue remodeling [60]. TGF-β binds receptors at the cell surface and activates Smad and non-Smad signaling pathways [61]. It was proved that TGF-β1 participates in the regulation of epithelial tight junction barrier and leads to disruptions in the epithelium integrity in ECRSwNP and noneosinophilic CRSwNP (NECRSwNP) [62].

Additionally, in Shin’s study PM significantly deteriorated RPMI 2650 cell viability and cytotoxicity. Macrophages treated with a conditioned medium from PM-treated nasal epithelial cells indicated increases in M1 macrophage-related markers (TNF-α, IL-1β, and IL-6) and a decrease in M2 macrophage-related marker (DC-SIGN). Shin et al. conducted a study that proved that PM treatments induced miR-19a and miR-614 expression in exosomes released from the human nasal epithelial cell line RPMI 2650 cells and hNECs. They confirmed that hNECs might promote proinflammatory M1 macrophages via upregulated miR-19a and miR-614 upon PM exposure. Furthermore, the purified exosomes increased in proinflammatory macrophage markers (TNF-α, IL-1β, and IL-6) in M0 macrophages, whereas this effect was blocked for the exosomes treated by RNAses [22].

Collectively, these results may imply that nasal epithelial cells affected by PM can release mi-R-19a and miR-614 via exosomes, which could lead to stimulating proinflammatory macrophage differentiation. Additionally, it was demonstrated that the downregulation of RORα expression, as a target molecule of miR-19a and miR-614, leads to M1 macrophage differentiation and an increase in proinflammatory cytokines. Shin et al. confirmed the above results in CRS and demonstrated that in tissues from CRSwNP, patients’ RORα expression is reduced, whereas miR-19a and miR-614 levels increased compared to normal tissue [22].

These studies demonstrate that miRNAs regulate immune functions and inflammation, are major drivers of cell fate specification and differentiation, and play a role in the control of CRS development, which means that the dysregulation of miRNA-mediated mechanisms is important in the pathophysiology of CRS [12,13,63,64].

### 3.8. EV-Mediated Fibroblast Interactions in CRS

Several studies focus on EV-mediated fibroblast interactions in CRS. Vascular endothelial growth factor (VEGF) is a signal protein with important proangiogenic activity, which has a mitogenic and anti-apoptotic effect on endothelial cells and increases vascular permeability [65]. The expression of VEGF and its receptors has been described as contributing to edema and angiogenesis in CRSwNP patients [66,67]. Nasal fibroblasts play an important role in the process of tissue remodeling and nasal polyp formation through myofibroblast differentiation, inflammatory cell infiltration by releasing cytokines and extracellular matrix (ECM) proteins including collagens, fibronectin, and vimentin [68,69,70]. VEGF is significantly higher expressed in CRSwNP patients relative to the CRSsNP group [71], and the expression of VEGF significantly increased in tissues from ECRSwNP patients compared to NECRSwNP and HS [72]. The local modulation of VEGF expression was suggested to be a potential therapeutic strategy in the management of CRSwNP [71]. In nasal polyp tissues, strong immunostaining for VEGF was found in the endothelium of blood vessels and the infiltrating perivascular inflammatory cells [66]. In polypous specimens from CRSwNP patients, the expression of p-STAT3 and VEGF and eosinophil infiltration significantly increased, and it was positively correlated [73]. In CRSwNP, upregulated VEGF expression causes angiogenesis and an increase in vascular permeability [74]. It was also shown that CRS patients with peripheral, mucus, or mucosal eosinophilia are likely to experience recurrence after endoscopic sinus surgery [75].

Shimizu et al. [21] conducted a study, which showed that interactions between peripheral blood eosinophils or EoL-1 cells and NPFs induced the release of exosomes and VEGF, but the release of exosomes was stimulated earlier (at 3 h of incubation) than VEGF’s release. In the same study, it was demonstrated that NPF-derived EV incubation with EoL-1 eosinophilic leukemia cells for 20 h resulted in the significant stimulation of VEGF release from EoL-1 cells, whereas cultured NPF-derived EVs alone did not produce VEGF for 24 h. Eosinophil-fibroblast interactions are significant in tissue remodeling in eosinophilic inflammation. NPF-derived EVs play a role in the release of VEGF, but they do not affect the release of VEGF from NPFs. The precise mechanism by which eosinophil-NPF interaction causes the release of exosomes from cocultured cells is obscure [21]. Furthermore, they also ascertained that under hypoxic conditions, exosome secretion from cultured NPFs was stimulated, and although the cause of this phenomenon is not fully known, it can suggest that cellular stresses may induce exosome secretion [21].

In another study, Shimizu et al. indicated that eosinophil–epithelial cell interactions significantly stimulated the secretion of mucin 5AC and VEGF and are important in tissue remodeling, which is present in chronic inflammation of paranasal sinuses [76]. It was investigated that in ECRS, eosinophil-derived osteopontin stimulates fibroblasts relative to the expression of IL-6, IL-8, and VEGF, which leads to myofibroblast differentiation and the overexpression of extracellular matrix (ECM) components [77].

Moreover, Wang et al. [23] demonstrated that treating CRSsNP-derived fibroblasts with labeled NLF-derived exosomes of CRSwNP patients led to the acquisition of labeled exosomes by fibroblasts, and the mucin 5AC protein carried by labeled exosomes was detected by Western blotting, which confirms mucin 5AC transfers by the NLF-EVS to recipient cells. Additionally, Wang et al. investigated the impact of the exosomal mucin 5AC, the major mucin in NM, on the recipient cells and determined significantly increased levels of COX-2, VEGF, and MMP-9, but not MMP-2, relative to cells cultured in exosome free media, which suggest that mucin 5AC might promote the production of MMP-9 and VEGF via the COX-2/PGE2 pathway [23]. This observation is in line with other studies that confirmed the significant increase in matrix metalloproteinase-9 (MMP-9) levels and a decrease in MMP-1 tissue inhibitor (TIMP-1 and TIMP-2) levels in nasal polyp specimens relative to mucosa from HS [78,79,80,81,82]. That suggests the role of those factors in nasal polyp formation. Additionally, it has been proven that IL-19 or IL-17A treatment significantly elevates the production of MMP-9 in hNECs [80,81], and cigarette smoking is associated with an increased expression of MMP-9 in the nasal tissues of patients with asthma and CRS [83].

In conclusion, the above-mentioned data suggest that EVs can stimulate VEGF and mucin 5AC release, which play a role in increased vascular permeability, angiogenesis, tissue remodeling, and nasal polyp formation. 

### 3.9. Immune Influence of CRS-Derived EV

Only one study demonstrated the effects of NLF-derived EVs on immune responses [8]. In an in vitro study, it was observed that nasal exosomes can induce a significant and dose-dependent migration of primary monocytes, NK cells, and neutrophils, which may indicate that nasal exosomes have a role in the recruitment of immune cells to the nose, which might be altered during inflammatory airway diseases. In Laser’s study [8], the findings revealed strong associations in terms of NLF-derived EVs with immune-related functions, such as immune cell trafficking, compared to other, previously published exosomal proteomes; many nasal exosomal proteins were associated with immune-related functions. Nasal exosomes contained 80 proteins classified as involved in the innate immune response, including the S100 proteins, induced nitric oxide synthase (NOS2), and BPIF proteins. A group of S100 proteins (S100A8, S100A9, and S100A12) has been proved to be consistently decreased in NLF exosomes collected from patients with asthma and CRS compared to healthy subjects and patients with asthma only, which might suggest impaired barrier function and increased susceptibility to bacterial and fungal overgrowth. Lasser et al. demonstrated the decreased expression of cathepsin G in NLF in the group of patients with asthma and CRS compared to only asthma patients, which could cause decreased antifungal response [8].

Different expression patterns of S100 proteins have been observed depending on subtypes of CRS, but further research focused on their role in mediating immunity is needed because those proteins are important in innate immune responses, epithelial barrier maintenance, and repair [84]. In one study, it was demonstrated that the levels of S100 A8/A9 protein decreased in NLF from CRSwNP and CRSwNP patients but simultaneously increased in nasal polyp tissue and correlated with levels of neutrophils [85]. In another study, tissue S100A8, S100A9, and heteromeric S100A8/A9 levels were significantly higher in CRSwNP, presenting increased depositions on extracellular matrix (ECM) structures with respect to the CRSwNP tissue [86]. Cho et al. observed a significant age-related decline in S100A8/A9 in nasal tissue samples from patients with CRSwNP [87]. Pulsipher et al. conducted a study, which showed that the tissue level of S100A12 was significantly elevated in CRSsNP patients relative to CRSwNP patients and HS, and the increased protein levels of S100A12 were significantly correlated to radiological disease severity [88]. Together, the data indicate that EVs carry chemical signals that direct the immune system.

### 3.10. CRS-Derived Evs Promote Angiogenesis and Vascular Permeability

Several studies demonstrated the effect of EVs on vascular and lymphatic endothelial cell lines, which was being investigated in CRS as well.

Zhang et al. showed that the incubation of fluorescent NLF-derived exosomes (NLFDEs) from CRSwNPs patients resulted in a transfer of fluorescence to human umbilical vein endothelial cells (HUVECs) and stimulated the tube formation, proliferation, and vascular permeability of HUVECs [25,26]. In Zhang’s study, miR-22-3p was highly expressed in NLF-derived exosomes from CRSwNP relative to the HS group, and it was demonstrated that the vascular permeability of HUVECs through the upregulation of miR-22-3p was enhanced, whereas it was inhibited by silencing miR-22-3p expression. In the same study, HUVECs cocultured with miR-22-3p-overexpressing exosomes increased vascular permeability. Zhang et al. used TargetScan to predict candidate targets of miR-22-3p. Vascular endothelial (VE) -cadherin expression was lower in tissue from CRSwNP patients in comparison to the HS group, whereas miR-22-3p was overexpressed in CRSwNP tissue samples. By inhibiting miR-22-3p, the expression of VE-cadherin in HUVECs increased. Zhang’s study also revealed that the permeability of HUVECs enhanced when VE-cadherin decreased. Results obtained in Zhang’s study suggest that exosomes contain and transfer miR-22-3p, which might influence vascular permeability by directly targeting VE-cadherin [26].

The potential therapeutic role of miR-22-3p was widely investigated and confirmed in the regulation of angiogenesis by targeting VE-cadherin [35,89,90,91,92,93,94,95].VE-cadherin is a component of adherents of vascular endothelial cells, which controls vascular permeability and inhibits unrestrained vascular growth [96]. The decreased expression of VE-cadherin is associated with endothelial paracellular permeability, which is observed in CRSwNP [97].

Another factor identified in CRS-derived EVs is a disintegrin and metalloprotease 10 (ADAM10), which impact angiogenesis and vascular permeability [98,99,100,101]. Zhang et al. confirmed that ADAM10 is overexpressed in NLF-derived exosomes in CRSwNP and promotes nasal polyp formation [25]. Nonetheless, it should be pointed out that in other studies, ADAM10 mRNA and protein levels in tissue do not differ significantly between nasal polyp tissue and nasal tissue from HS groups [102,103]. Zhang et al. presented that NLF-derived exosomes from CRSwNP can promote angiogenesis and vascular permeability, which might be related to the overexpression of ADAM10 [25].

### 3.11. Exosomal Coagulation Pathway Derangement in CRSwNP

The upregulation of the coagulation system as involved in the pathogenesis of tissue remodeling in CRSwNP has been investigated [104,105,106,107,108]. 

Mueller et al. [17] found that coagulation pathway tissue proteins were most significantly associated with CRSwNP relative to all other pathways identified by IPA, and in MetaCore^TM^ analyses, 13 were upregulated and 1 (von Willebrand factor) was downregulated, whereas the majority of fibrinolysis-associated tissue proteins, including plasmin and tissue-type plasminogen activator (tPA), were downregulated in CRSwNP relative to HS. Among the 13 significantly altered coagulation-related tissue proteins, fibronectin and fibrinogen γ chains were the most overexpressed in CRSwNP compared to the control group. Furthermore, it was demonstrated that the exosomal proteome exhibited an inverse, strong, and highly statistically significant correlation with the matched tissue proteome, which hypothetically could be due to the depletion of cellular protein after packaging and release into the exosomes. In the same study, overexpressed plasma kallikrein and vitamin K-dependent protein S and downregulated coagulation factor IXab were described as novel potential tissue biomarkers for CRSwNP. Moreover, the authors noticed that the plasmin inhibitor, α-2-antiplasmin (serpinF2) was upregulated in CRSwNP, which interfered with fibrinolysis [17]. Later, they confirmed this observation in the next study [16], where they conducted nasal tissue transcriptome analysis for serine protease inhibitors (serpinB2, serpinE1, serpinF2, and serpinG1) followed by validation by an analysis of Western blots from nasal tissue and NM exosomes. They found a strong and significant correlation among the selected genes and between tissue and exosomes. Tissue and exosomal expressions of serpinB2, serpinE2, serpinF2, and serpinG1 were higher among the CRSwNP patients relative to the HS group. IHC showed different localization and staining intensity for SerpinB2, SerpinE1, SerpinF2, and serpinG1 in CRSwNP and control patients.

Exosomal Serpine 1 was also identified by Zhou as differentially expressed in CRSwNP than in the HS group [27]. The study conducted by Zhou also showed that hNECs-derived exosomes in CRSwNP patients contain proteins that are involved in the epithelial remodeling of the airway via p53 and PPAR signaling pathways [27]. Serpine 1 was presented as involved in CRS and AR, and it is considered to play a role in tissue remodeling [109].

Workman et al., using a bioinformatic tool, demonstrated that blood coagulation (GO:0030195) and fibrinolysis regulation (GO:0051917) pathways were significantly upregulated in exosomes from CRSwNP patients and decreased with steroid treatment, which might be a piece of evidence for its importance in the mechanism of action of steroids in CRSwNP [24]. In CRSwNP, profound fibrin deposition was described, which may have a role in tissue remodeling and nasal polyp formation [106]. It might be related to increased levels of thrombin/antithrombin complex (TATc) and thrombin activatable fibrinolysis inhibitor (TAFI) observed in NLF of CRSwNP patients [110]. Activated coagulation factors, thrombin, and FXa were shown to significantly stimulate the release of TGFbeta1, fibronectin, eotaxin-1, IL-6, and IL-8 from NPFs in CRSwNP [69].

### 3.12. Effect of the Diseased Epithelial Exosomes on the Proliferation of hNECs

Zhou et al. [27] investigated the effects of NLF-derived and hNECs-derived exosomes from CRSwNP and CRSwNP and asthma patients on normal hNECs proliferation and found a significant reduction in the rate of proliferation of normal hNECs after 6–7 days from adding NLF-EVs and after 4–5 days from adding hNECs- derived Evs. Then, Zhou et al. determined the effective concentration of hNECs-derived exosomes in inducing reduced epithelial proliferation in CRSwNP patients and CRSwNP and the asthma group, which was 10 µg/mL or greater. Those findings may indicate that CRSwNP exosomal proteins inhibit adjacent epithelium growth. Zhou et al. also tested the effects of diseased exosomes on the normal differentiation of hNECs but did not detect effects affecting the expression of ciliary markers (forkhead box protein J1 and dynein axonemal heavy chain 5) and the goblet cell marker—mucin 5AC [27].

### 3.13. Exosomal Biomarkers Predicting Presence, Phenotype, and Disease Severity in CRS

The presence of evident changes in EVs and protein expression in NM suggests these factors could be used to predict disease severity and treatment response.

Mueller et al. [15] presented exosomal proteomic biosignatures predicting CRSwNP, among which are cystatin SN (CST-1), peroxiredoxin-5 (PRDX5), and platelet glycoprotein VI (GP6). The authors validated this proteomic data set by performing Western blots on independent samples of tissue and exosomes in the CRSwNP and HS groups and confirmed that CST-1, PRDX5, and GP6 were significantly differentially expressed in both tissue and exosomes among the CRSwNP group relative to HS group [15]. Peroxiredoxin-5 (PRDX5) might trigger a proinflammatory response by inducing the expression of proinflammatory cytokines in macrophages through activation of Toll-like receptors [111,112]. The activation of toll-like receptor 4 (TLR4) is important for initiating allergic inflammation and antifungal immunity [113]. Platelet collagen receptor glycoprotein VI (GPVI) is implicated in platelet activation and aggregation [114] and was also presented as contributing to local host defense during pneumonia-derived sepsis [115], which suggests that it might be a potential therapeutic target in inflammatory diseases.

Cha et al. showed that the mucin-type O-glycan biosynthesis was a high-ranked predicted pathway in the presence of CRS, while transforming growth factor beta β (TGF- β) signaling pathway was a high-ranked predicted pathway in CRSwNP relative to CRSsNP patients [12].

Nocera et al. proved that the median concentration of exosomal permeability-glycoprotein (P-gp), a transmembrane glycoprotein, was significantly enriched among CRSwNP patients relative to the HS group, but earlier, it was also determined that the mucus concentration of P-gp is significantly higher in CRS, particularly in CRSwNP [28]; therefore, both methods could be used noninvasively to determine the level of this biomarker. P-gp is an epithelial driver of type 2 inflammation [116,117], and its expression in SM increases after CST-1 exposure [20]. Furthermore, Nocera’s study was the first to present an epithelial transfer of autologous exosomes and their protein cargo, P-gp, within primary human samples [19]. P-glycoprotein (P-gp) is an ATPase transporter, which is upregulated in CRSwNP and associated with eosinophilic inflammation and mometasone resistance [66,67]. The use of P-glycoprotein inhibitors in CRS can be considered, but more research is needed to confirm its safety and efficacy [118,119,120,121,122].

In Nocera’s study, CST-1 and CST-2 (members of the type 2 cystatin proteins superfamily) were among the most overexpressed proteins in the mucosa, mucus, and mucus-derived exosome samples in CRSwNP patients relative to HS. The author showed that exosomal CST-1 and CST-2 were strongly and significantly correlated with tissue eosinophil per HPF, Lund–Mackay-computed tomography scores, and 22-Item Sino-Nasal Outcome Test scores (SNOT-22). CRSwNP patients with allergy or aspirin-exacerbated respiratory disease (AERD) demonstrated a trend towards increased exosomal CST-1 and CST-2 expression in comparison to CRSwNP without coexisting allergy and AERD [20]. In Nocera’s study, an exposure to CST-1 preferentially induced the production of Th2 cytokines (IL-4, IL-5, and IL-13), IL-6, eotaxin, and P-gp expression in mouse septonasal tissue when compared with the baseline, particularly after 18 days of CST-1 treatment. After establishing the recombinant CST-1 mouse model, Nocera et al. caused the knockdown of ABCB1a, which encodes P-glycoprotein (a regulator of epithelial cytokine secretion), and achieved a reduction in CST-1-induced type 2 cytokine expression (IL-4, IL-5, IL-10, and IL-13) in a dose-dependent manner when compared with baseline CST-1 [20].

Miyake et al. [14] collected data concerning values of exosomal cystatins and determined that the expression of CST-1 and CST-2 in CRSwNP was significantly higher than in CRSsNP patients.

Other authors found that CST-1 is highly expressed in nasal polyp tissue from patients with ECRSwNP compared with NECRSwNP patients, suggesting that CST-1 might contribute to the severity and recurrence of CRSwNP [123]. In Yan’s study, tissue CST-1 expression significantly increased in ECRSwNP patients (especially with coexisting asthma and correlated with eosinophilia in tissue) but decreased in NECRSwNP relative to HS. Additionally, in the same study, it was presented that the incubation of dispersed nasal polyp cells with recombinant CST-1 significantly upregulated the expression of IL-5 [124], which corresponds to Nocera’s results [20]. It was demonstrated that in CRSwNP’s high concentration of cystatin NP in nasal secretions is correlated with a faster onset and higher rate of uncontrolled status [125]. CST-2 levels in mucus samples were investigated as predictors for early recurrences and resistance to treatments in CRS [126].

Zhou et al. analyzed hNECs-derived exosomes of the CRSwNP with coexisting asthma patients and found increased levels of the innate immune response in mucosa and glycerolipid and glycerophospholipid metabolism, suggesting that it might be involved in poor prognosis.

### 3.14. Therapeutic Influence on CRS-EVs

EVs have been highlighted as a factor of drug action, and their inhibition might be a therapeutic target in airway inflammation. On the other hand, they have also been shown to be potential carriers for targeted drug delivery.

To determine whether NPFs-derived exosomes are involved in VEGF’s production in cocultured cells, Shimizu et al. used GW4869 and DMA, known for reducing the number of exosomes released [127,128,129], for the pretreatment of NPFs. They achieved a reduction in the release of exosomes and VEGF from cocultured EoL-1 cells and NPFs. This may potentially imply that exosome inhibition might be a therapeutic target in CRS [21].

Workman et al. [24] analyzed exosome samples from the mucus of CRSwNP patients utilizing the SOMAscan platform before and after a 16-day taper oral prednisone course and determined how differentially expressed proteins are affected as a result of treatment. Based on previous work concerning proteomic analysis in CRSwNP [130], authors identified 18 proteins demonstrating significantly (at least 2-fold) lower concentrations in CRSwNP, of which 16 had an average positive change after steroid treatments. Proteomic analyses were proposed as effective tools for analyzing the therapeutic effects of steroid therapy in CRSwNP and, perspectively, to find more precise treatment methods [24].

The most significantly decreased protein in CRS in this study was lactoperoxidase, which demonstrated a two-fold increase in concentration after steroid therapy, which can suggest the importance of this protein in CRS. Lactoperoxidase is known as a natural antibacterial agent and has been demonstrated to have a role in the scavenging of hydrogen peroxidase in asthmatic airways [131]. In Workman’s study [24] bactericidal/permeability-increasing protein (BPI) were found at increased concentrations in exosomes from CRSwNP patients and further increased after steroid treatments, but another study demonstrated the reduced expression of BPI in tissue from CRSwNP patients [132]. Workman et. al. also identified 53 proteins having significantly (at least two-fold) higher concentrations in CRSwNP before the steroid course, but the next analysis showed that only 42% (22 proteins) had an average decrease in concentration after the oral steroid course, whereby a few increased proteins (apolipoprotein L1, tenascin, casein kinase II subunit α, angiogenin, and bactericidal/permeability-increasing protein) showed increases of >100% after steroid treatment [24].

### 3.15. Discussion

CRS is characterized by chronic inflammation of the SM, which is divided clinically into CRSwNP and CRSsNP. Progress in our understanding of CRS pathophysiology has led to the adoption of the endotype paradigm of disease characterization. The main goal of CRS research is to understand its etiopathology and, as a consequence, to improve preventative strategies, develop diagnostic tools, and design personalized therapies. It is hypothesized that exosomes in the nasal cavity have biological functions and that their molecular components are changed among individuals with CRS.

In recent years, there has been increasing interest in research regarding the mechanisms and roles of exosomes in different diseases. This review focuses on the current state of knowledge on the role of exosomes in CRS pathophysiology. Although exosomes were discovered almost 40 years ago [133,134], their meaning in CRS is still not sufficiently elucidated and utilized. It is also worth noting that there are differences in the abundance and morphology of exosomes and expression of exosomal proteins and miRNA in CRS.

The isolation of exosomes from NLF or mucus in diagnostics of CRS can be a good alternative for tissue sampling because this method is effective, minimally invasive, easy, and possible to carry out without anesthesia and damage to the mucosa [24]. It should be noted that the most common technique of exosome isolation is UC [9,10,33,34,135], but this method is time-consuming and requires costly equipment [136], even though the content of proteins in the entire NM was the subject of studies concerning CRS [137,138,139,140,141]. Whole mucus does not reflect underlying tissue proteomes, as well as exosomal proteins, which are protected from degradation by nucleases and proteases, and their analysis is more reflective of the protein milieu of the host cell [15]. It was already shown that NLF is poorly quantitatively representative of nasal polyp tissue protein level [142].

This study systematically analyzed changes in the quantitative and qualitative production of exosomes in CRS and the significance of the abnormal expression of exosomal biomarkers in CRS. It is known that exosomal cargo may play a role in immune response, angiogenesis, vascular permeability or coagulation, and fibrinolysis pathways, which was presented above. Additionally, Nocera et al. provided in vivo and in vitro evidence for an increase in NM-derived exosome secretion and exosomal inducible nitric oxide synthase expression triggered by lipopolysaccharide, which is a novel exosome-mediated defense mechanism [143]. Nevertheless, intraepithelial communication via exosomes is not completely clarified, because it is still unknown which cargo ends up in the exosome that is transported to other cells [136].

MiRNAs are already known to be involved in the regulation of the inflammatory response in various human cells. Recently, there is growing evidence of EV miRNA’s role in CRS development [12]. MiRNA is relatively stable as opposed to mRNA and protein and might be a noninvasive biomarker in CRS [12]. It has been already demonstrated that exosomes can efficiently deliver miRNA to breast cancer cells [144]. Understanding the potential of NLF- or NM-derived exosomes in the diagnostics and treatment of CRS is still ahead of us, but we can see from the example of other liquid biopsies that this direction can bring along tremendous possibilities. For example, EGFR testing in EVs isolated from bronchoalveolar lavage fluid (BALF) was shown as a potential diagnostic method in advanced non-squamous non-small-cell lung carcinoma, which can be used alternatively for lung tissue biopsy [145]. The therapeutic potential of exosomes in CRS has not been fully characterized, and further studies are needed.

Different proteins and miRNA were investigated in included studies as exosomal cargo in CRS and many of them may be important in the diagnosis of CRS. The most promising biomarkers seem to be CST-1 and CST-2, mucin 5AC, P-gp, and, among miRNAs, miRNA-19a, miRNA-614, and miRNA-22-3p. The role of those biomarkers needs further exploration. Hopefully, in future, it would allow for using them in daily clinical routines to predict disease severity and response to treatment. It is worth mentioning, that noninvasive exosomal sampling could be performed in an outpatient setting.

Nasal exosomes have a role in immune-related functions in CRS and contain proteins involved in the immune response [8], and they are important in coagulation and fibrinolysis regulation [16,17,24] and can stimulate vascular permeability and angiogenesis [25,26]. There is a need for further research in CRS on angiogenesis, vascular permeability, coagulation, and fibrinolysis regulation pathways because it can move us closer to new therapeutic options for CRS patients. The transfer of fluorescence through NLF-derived EVs was confirmed [25], which suggests that exosomes can be used for transferring drugs to recipient cells.

In this review, we only presented studies concerning human cell-derived exosomes. However, it is interesting that bacteria also can secrete EVs, containing biological information such as proteins, nucleic acids, and lipids, that can be delivered to recipient cells. Choi et al. demonstrated that CRS patients have greater bacterial abundance and decreased diversity in bacterial compositions in NLF relative to HS. Bacteria composition is positively correlated with the composition of EVs secreted from microbiota and *Staphylococcus aureus* and its EV compositions are higher in samples from CRSwNP patients relative to CRSsNP [146]. *Staphylococcus aureus* EV can induce neutrophilic inflammation in respiratory airways and enhance the development of airway hypersensitivity relative to inhaled allergens [147]. In Kim’s study, some sinus bacteria and serum bacterial EVs showed positive correlations [148].

The findings included in this review studies are somewhat limited by the relatively low number of patients from whom the samples were obtained, especially when we take into account the heterogeneity of CRS and interpatient variability associated with endotypes of CRS, previous therapies, comorbidities, individual predispositions, and environmental factors. Small sample sizes may lead to an overestimation of observed effects and allow only for the qualitative analysis of exosomal proteins. Further research on larger sample sizes is required to confirm obtained data. Only part of the papers classified CRS patients into eosinophilic and non-eosinophilic endotypes, which may demonstrate differences in exosomal cargo, both proteins, and miRNA. There are many differences in methods used in included studies, which poses a major challenge in terms of interpretation. There might be some inconsistencies related to this when comparing different trials. A weakness of Nocera’s research is a lack of the possibility of distinguishing whether the enhanced P-gp function came from P-gp transfers from exosomes or an increase in P-gp activity [28]. A limitation of Shimizu’s article is the number of cultured blood eosinophils, which was ten times lower than EoL-1 cells. NPF-derived EVs stimulated VEGF release from EoL-1 cells but not from blood eosinophils. Another limitation of that study was that the proportion of exosomes released from NPFs and eosinophils/EoL-1 cells was unclear [21]. In Nocera’s research, it was confirmed in a murine model, CST1 can induce type 2 inflammation, but clinical studies are necessary to confirm this mechanism in CRS patients [20]. Finally, we have to remember that in vitro study results are not always parallel to clinical results. This is why some results should be interpreted with caution.

A great benefit of included papers is that they are the first to present the ability to isolate and analyze nasal exosomes. All articles transparently report experimental details and are innovative in presenting the roles of exosomes in CRS. It is worth pointing out that Lasser’s study included the first description of the proteome of nasal exosomes [8] and a few other papers showed a correlation between the tissue and exosomal proteomes, which can contribute to using less invasive methods in research on CRS [16,17,18]. The strength of several included studies includes providing different pathway derangements in CRS and finding novel biomarkers that could be used for CRS severity and treatment prediction, which was possible by using a bioinformatics analysis of proteomic data [12,13,24,27]. An additional asset is that in a few included papers, SOMAscan proteomic analysis was demonstrated, which allows the study of exosomal proteomics from the low sample volume [15,17,18,20,24]. It was also presented that using novel bioinformatics enables the identification CRS clusters that are consistent in terms of disease severity [14]. The merit of Shimizu’s study is that it is the first to demonstrate eosinophil–fibroblast interactions [21]. The big advantage is that included papers provide novel information about CRS pathophysiology and provide an avenue for further research on exosomes to study immunological mechanisms observed in CRS.

The greatest advantage of this review is the fact that it contains the most up-to-date and extensive systematic summary of the available data on the role of exosomes in CRS. Hopefully, this paper will contribute to expanding the knowledge on the potential of using exosomes and can affect a larger quantity of research on this topic, which will allow us to better understand the pathophysiology of CRS and provide diagnostics and therapy tailored to suit each patient.

## 4. Conclusions

Presented findings indicate that exosomal proteins and miRNA levels can be tested by conducting a non-invasive, highly available liquid biopsy. NM or NLF may be easily obtained as an outpatient. Exosomes reflect tissue proteomes and allow the study of proteomic alterations in CRS instead as a substitute for tissue sampling. Exosomal biomarkers can be used to specify presence and phenotypes and predict disease severity, increased recurrence rates, and treatment responses. The therapeutic potential of exosomes in CRS has not been fully characterized and further studies may contribute to finding novel therapeutic targets. The first steps have already been taken, but more advanced research on nasal exosomes is needed, which might open a wider door for individualized medicine in CRS.

## Figures and Tables

**Table 1:** The inclusion and exclusion criteria.

	Inclusion Criteria	Exclusion Criteria
** Study status **	Completed, Published	Unfinished, Unpublished
** Study type **	Original article	Reviews Book chapters Expert opinions Conference papers Case reports
** Language **	English	Other than English
** Sample origin **	Exosomes from human cells	Bacteria-derived exosomes
** Topic **	Studies concerning CRS	Studies not related to the CRS topic
** Quality **	Good quality of research studies	Poor quality of research studies

**Table 2:** Characteristics of studies included in the systematic review.

Study	The Source of Exosomes	Study Group	Control Group	Exclusion Criteria	Main Results
**Laesser et al. (2016)** [8]	• NLF	• asthma (n = 15) • asthma with coexisting CRS (n = 13)	• HS (n = 14)	• Exposition to antihistamines for 72 h, LABA for 24 h, SABA for 8 h and Spiriva for 24 h	• Nasal exosomes induced the migration of several immune cells (monocytes, neutrophils, and NK cells). • 604 proteins were identified in nasal exosomes. • The nasal exosomal proteome showed strong associations with immune-related functions, such as immune cell trafficking. • In the exosomes from patients with respiratory diseases serum-associated proteins and mucins were upregulated. In contrast, proteins with antimicrobial functions and barrier-related proteins were downregulated relative to the control group.
**Nocera et al. (2017)** [19]	• NM	• CRSwNP (n = 10)	• HS (n = 10)	• Exposition to antibiotics or steroids in last 4 weeks, • Ciliary dysfunction, autoimmune disease, CF, immunodeficiency, smoking	• Exosomal P-gp was significantly enriched among the CRSwNP relative to the control group. • Exosomes were absorbed by cultured sinonasal epithelial cells within 10 min leading to a significant increase in P-gp activity in CRSwNP patients in comparison to the control group.
**Zhang et al. (2018)** [25]	• NLF	• CRSwNP (n = 25)	• HS (n = 15)	• Exposition to topical /systemic steroids in last 3 weeks, • Allergic rhinitis, asthma, aspirin sensitivity	• Incubation of fluorescent NLF-derived exosomes resulted in transferring of fluorescence to HUVECs. • NLF-derived exosomes from CRSwNP patients stimulated tube formation, proliferation, and permeability of HUVECs. • ADAM10 was enriched in NLF-derived exosomes from CRSwNP patients relative to the HS group.
**Mueller et al. (2018)** [17]	• NM	• CRSwNP (n = 10)	• HS (n = 10)	• Exposition to antibiotics or any topical /systemic steroids in last 4 weeks • Ciliary dysfunction, autoimmune disease, CF, immunodeficiency • Among the controls additionally allergy or asthma	• Among all protein pathways, the coagulation cascade was most significantly associated with CRSwNP. • The correlation between tissue- and NM-derived exosomal fibrinolysis and coagulation protein expression was strong, inverse, and highly significant. • Novel tissue proteome findings: overexpression of plasma kallikrein and vitamin K-dependent protein S and the downregulation of coagulation factor IXab in CRSwNP.
**Mueller et al. (2019)** [15]	• NM	• CRSwNP (n = 20)	• HS (n = 20)	• Exposition to antibiotics or any topical /systemic steroids in last 4 weeks • Ciliary dysfunction, autoimmune disease, CF, immunodeficiency • Among the controls additionally allergy or asthma	• The exosomal proteome demonstrated 123 significantly differentially regulated proteins in CRSwNP relative to HS. • 80 exosomal proteins overlapped with the matched CRSwNP tissue proteome, whereas 4 proteins overlapped with the matched whole mucus samples. • 43 significantly dysregulated pathway networks overlapped between the exosomal and tissue proteome in CRSwNP, whereas only 3 among them matched withwhole mucus samples.
**Mueller et al. (2019)** [16]	• NM	• CRSwNP (n = 16)	• HS (n = 16)	• Exposition to antibiotics or any topical /systemic steroids in the last 4 weeks • Ciliary dysfunction, autoimmune disease, CF, immunodeficiency, • Among the controls additionally allergic rhinitis, AERD and asthma	• Exosomal and tissue expression of serpinB2, serpinE1, serpinF2 and serpinG1 was higher in CRSwNP patients than in HS group. • There was a strong and significant correlation among the serpinB2, serpinE1, serpinF2, and serpinG1 genes for tissue and exosomes.
**Miyake et al. (2019)** [14]	• NM	• CRSwNP (n = 40) • CRSsNP (n = 33)	• HS (n = 32)	• Ciliary dysfunction, autoimmune disease, CF, immunodeficiency • Among the controls additionally current smoking or asthma	• Expression of exosomal CST-1 was significantly higher in CRSwNP and control group than in CRSsNP group. • Expression of exosomal CST-2 was significantly higher in CRSwNP than in both the CRsNP and control groups. • Seven clusters were identified among patients based on cystatin expression, diagnosis, demographic, and biological variables. CST-2 levels trended linearly with phenotype and clinical severity parameters.
**Zhou et al. (2020)** [27]	• NLF • hNECs from NM	• CRSwNP without coexisting asthma (n = 3) • CRSwNP with coexisting asthma (n = 3)	• HS (n = 3)	• Exposition to leukotrienes and antibiotics • Atopy, aspirin and nonsteroidal anti-inflammatory drug intolerance • Among the controls, there is additional exposition to topical /systemic steroids	• The hNECs-derived exosomes from patients with CRSwNP with and without asthma contained differentially expressed proteins that were mainly involved in epithelial remodeling via pathways such as p53. • Epithelial-derived exosomes from CRSwNP patients (with and without coexisting asthma) significantly reduced the proliferation rate of control hNECs at an effective concentration of ≥10 µg/mL.
**Workman et al. (2020)** [24]	• NM	• CRSwNP (n = 12)	-	• Exposition to oral steroids or antibiotics in last 30 days • Ciliary dysfunction, autoimmune disease, CF, immunodeficiency	• 18 proteins were identified to be highly underexpressed in CRSwNP, of which 16 increased after steroid treatment, including lactoperoxidase and platelet factor 4. • 53 proteins were highly overexpressed in CRSwNP, of which 22 decreased in quantity after steroid treatment. • Blood coagulation and fibrinolysis regulation pathways of the NM were significantly upregulated in exosomes from CRSwNP patients and decreased after the oral prednisone course.
**Mueller et al. (2020)** [18]	• NM	• CRSwNP (n = 46)	• HS (n = 46)	• Exposition to antibiotics or any topical /systemic steroids in last 4 weeks • Ciliary dysfunction, autoimmune disease, CF, immunodeficiency • Among the controls additionally allergy or asthma	• Significant upregulation of tissue and exosomal PAPP-A on a proteomic, transcriptomic and functional level in CRSwNP compared to HS group. • The transcriptomic data using qPCR demonstrated a significant upregulation of PAPP-A in CRSwNP, a significant downregulation of the inhibitor stanniocalcin-1 (STC-1), STC-2 and insulin-like growth factor binding protein–5 (IGFBP-5) and no differences for IGFBP-4 and insulin-like growth factor-1 (IGF-1) between the CRSwNP and HS group.
**Shin et al. (2020)** [22]	• cultured human nasal epithelial cell line RPMI 2650	• NECRSwNP (n = 9) • RPMI 2650 cell culture treated with PM for 48 h • PM-treated hNECs	• HS (n = 9) • RPMI 2650 cell culture not treated with PM • control hNECs	• Exposition to oral or topical medication (including steroids, antihistamines, antibiotics) in last 3 months	• The induction of exosomal miRNAs from hNECs upon airborne PM exposure promotes proinflammatory M1 macrophage polarization via downregulation of RORα expression in the human respiratory mucosal microenvironment. • Exosomal miRNA-19a and miRNA-614 directly bind to the 3′-untranslated region of RORα mRNA and downregulate RORα expression, which leads to inflammation due to inflammatory cytokine upregulation and M1/M2 macrophage polarization. • Enhanced expression of miRNA-19a and miRNA-614 and reduced RORα expression were observed in tissue from CRS patients relative to HS.
**Zhang et al. (2020)** [26]	• NLF	• CRSwNP (n = 26)	• HS (n = 10)	• Exposition to topical /systemic steroids in last 3 weeks • Ciliary dysfunction, CF, autoimmune disease, immunodeficiency	• MiRNA-22-3p was upregulated in NLF-EVs from CRSwNP relative to the HS group. • Exosomal miRNA-22-3p derived from CRSwNP enhanced the tubule permeability of HUVECs. • VE-cadherin was determined as a direct target of miRNA-22-3p. • MiRNA-22-3p regulated vascular permeability by targeting VE-cadherin in HUVECs.
**Cha et al. (2021)** [12]	• NLF	• CRSwNP (n = 7) • CRSsNP (n = 8)	• HS (n = 7)	NR	• The expression of exosomal miRNA was significantly increased in NLF of CRS patients relative to HS group. • 12 miRNAs were differentially expressed in exosomes from CRS patients relative to HS individuals, including seven upregulated miRNAs and five downregulated miRNAs. • 8 miRNAs were differentially expressed in the NLF-derived exosomes of CRSwNP relative to CRSsNP patients. • The mucin-type O-glycan biosynthesis was a high-ranked predicted pathway in the presence of CRS, while TGF- β signaling pathway was a high-ranked predicted pathway in CRSwNP relative to CRSsNP patients.
**Wang et al. (2021)** [23]	• NLF	• CRSwNP (n = 20) • CRSsNP -derived fibroblasts treated with the mucin 5AC-enriched exosomes	• CRSsNP (n = 22) • CRSsNP -derived fibroblasts treated with the mucin 5AC-deificent exosomes	• Exposition to topical /systemic steroids or antibiotics in last 2 weeks • Malignancies, asthma, upper respiratory tract infection within 4 weeks • Previous nasal surgery	• Mucin 5AC was significantly upregulated in NLF-derived exosomes of CRSwNP patients. • The expression of mucin 5AC was increased in the tissue specimens of the CRSwNP patients. • CRSsNP-derived fibroblasts treated with the mucin 5AC-enriched exosomes had a significantly increased level of VEGF, COX-2, and MMP-9 but not MMP-2 in comparison to those treated with mucin 5AC-deficient exosomes.
**Shimizu et al. (2022)** [21]	• NPFs cultured from nasal polyp tissue specimens	• ECRSwNP (n = 3) • NPFs incubated with GW4869 and DMSO • NPFs incubated with DMA and DMSO	• NPFs incubated with DMSO	NR	• Interaction of NPFs and peripheral blood eosinophils or EoL-1 cells stimulated the release of exosomes and VEGF. • NPF-derived EVs significantly stimulated VEGF release from EoL-1 cells, whereas cultured NPF-derived EVs alone did not produce VEGF for 24 h. • Pretreatment of NPFs with GW4869 or DMA attenuated the release of exosomes and VEGF from cocultured EoL-1 cells and NPFs.
**He et al. (2022)** [13]	• plasma	• CRSwNP (n = 5)	• HS (n = 5)	• Exposition to antibiotics, systemic /topical steroids or antileukotrienes in last 3 months • Among the controls additionally allergy or asthma	• 1692 known miRNAs and 1068 novel miRNAs were identified in plasma-derived exosomes. • 159 plasma exosomal miRNAs were differentially expressed (93 upregulated and 66 downregulated) by miRNA sequencing in CRSwNP relative to the HS group. • The top three upregulated miRNAs were novel_miRNA_677, novel_miRNA_1037, and novel_miRNA_79. • The top three downregulated miRNAs were novel_miRNA_192, novel_miRNA_1022, and novel_miRNA_4. • Corresponding differentially expressed target genes in the GO and KEGG analyses revealed: axon guidance, extracellular matrix (ECM)-receptor interaction, protein digestion and absorption, the calcium, the Hippo, the Notch, the ErbB, the cAMP signaling pathway, and focal adhesion.
**Nocera et al. (2022)** [20]	• NM	• CRSwNP (n = 10) • the recombinant CST-1 mouse model with ACSC1a knockdown (1 mg/kg or 0.1 mg/kg)	• HS (n = 10) • the recombinant CST-1 mouse model without ABCB1a knockdown	• Exposition to antibiotics or any topical /systemic steroids in last 4 weeks • Ciliary dysfunction, autoimmune disease, CF, immunodeficiency • Among the controls additionally the presence of environmental allergy or asthma	• CST-1 and CST-2 are among the most overexpressed protease inhibitors in tissue, mucus-derived exosome and mucus samples in CRSwNP patients in relation to HS. • Exosomal CST-1 and CST-2 were strongly and significantly correlated with tissue eosinophils and Lund-Mackay scores. • Exposure to CST-1 induced type 2 cytokine secretion and was abrogated by epithelial knockdown of ABCB1a, which encodes P-glycoprotein.

ADAM 10—a disintegrin and metalloprotease 10; AERD—aspirin-exacerbated respiratory disease; CF—cystic fibrosis; CRSwNP—chronic rhinosinusitis with nasal polyps; CRSsNP—chronic rhinosinusitis without nasal polyps; CST-1—cystatin-1; DMA—amiloride hydrochloride; DMSO—dimethyl sulfoxide; ECRSwNP—eosinophilic chronic rhinosinusitis with nasal polyps; GO—Gene ontology; HS—healthy subjects (without chronic rhinosinusitis); HUVECs—human umbilical vein endothelial cells; KEGG—Kyoto Encyclopedia of Genes and Genomes; LABA—long-acting β-agonist; miRNA—microRNA; NK cells—natural killer cells; NLF—nasal lavage fluid; NM—nasal mucus; NECRSwNP—noneosinophilic CRSwNP; NPFs—nasal polyp fibroblasts; NR—not reported; pHNEs—primary human nasal epithelial cells; P-gp—permeability-glycoprotein; PM—particulate matter; RORα—retinoic acid-related orphan receptor; SABA—short-acting β-agonist; TGF- β—transforming growth factor beta β; VE-cadherin—vascular endothelial-cadherin.

## Data Availability

Not applicable.

## Supplementary Materials

The supporting information can be downloaded at: www.mdpi.com/article/10.3390/ijms231911284/s1.
