# Supplementary material for: Roles of Exosomes in Chronic Rhinosinusitis: A Systematic Review
Source: Int J Mol Sci. 2022 Sep 25;23(19):11284. doi: 10.3390/ijms231911284 (PMC9570170; doi:10.3390/ijms231911284)
Supplement: Supplementary file 1 [file ijms-23-11284-s001.zip › Table S1. The search strategy for each database..pdf]

Table S1. The search strategy for each database.

**Pubmed**
(("Exosomes"[Mesh]) OR (exosome) OR (extracellular vesicle*) OR (exosomal) OR (liquid biopsy)) AND (("Sinusitis"[Mesh]) OR rhinosinusitis)
**Scopus**
TITLE-ABS-KEY ((exosome*) OR (extracellular vesicle*) OR (exosomal) OR (liquid biopsy)) AND ((sinusitis) OR rhinosinusitis)
**Cochrane**
#1 "Exosomes"[Mesh]<br>#2 (exosome) OR (extracellular vesicle*) OR (exosomal) OR (liquid biopsy)<br>#3 ("Sinusitis"[Mesh])<br>#4 (rhinosinusitis)<br>(#1 OR #2) AND (#3 OR #4)
**Web of Science**
(AB=((exosome*) OR (extracellular vesicle*) OR (exosomal) OR (liquid biopsy))) AND (AB=((sinusitis) OR (rhinosinusitis)))
