# Supplementary material for: Roles of Exosomes in Chronic Rhinosinusitis: A Systematic Review
Source: Int J Mol Sci. 2022 Sep 25;23(19):11284. doi: 10.3390/ijms231911284 (PMC9570170; doi:10.3390/ijms231911284)
Supplement: Supplementary file 1 [file ijms-23-11284-s001.zip › Table S2. Risk of bias assessed using OHAT risk of bias rating tool for human and animal studies..pdf]

Table S2. Risk of bias assessed using the OHAT risk of bias rating tool for human and animal studies.

	**Laesser [8]**	**Nocera [19]**	**Zhang [25]**	**Mueller [17]**	**Mueller [15]**	**Mueller [16]**	**Miyake [14]**	**Zhou [27]**	**Workman [24]**	**Mueller [18]**	**Shin [22]**	**Zhang [26]**	**Cha [12]**	**Wang [23]**	**Shimizu [21]**	**He [13]**	**Nocera [20]**
Was administered dose or exposure level adequately randomized?	(-)	(-)	(-)	(-)	(-)	(-)	(-)	(-)	(-)	(-)	(-)	(-)	(-)	(-)	(-)	(-)	(-)
Was allocation to study groups adequately concealed?	(- -)	(- -)	(- -)	(- -)	(- -)	(- -)	(- -)	(-)	(-)	(- -)	(-)	(-)	(-)	(-)	(--)	(-)	(-)
Did selection of study participants result in appropriate comparison groups?	(+)	(+)	(+)	(+)	(+)	(+)	(-)	(+)	(+)	(+)	(+)	(+)	(+ +)	(+ +)	(+)	(+ +)	(+)
Were experimental conditions identical across study groups?	(+ +)	(+ +)	(+)	(+)	(+)	(+)	(+)	(+)	(+ +)	(+ +)	(+ +)	(+ +)	(+ +)	(+)	(+ +)	(+ +)	(+ +)
Were the research personnel and human subjects blinded to the study group during the study?	(+)	(+)	(+)	(+)	(+)	(+)	(+)	(+)	(+)	(+)	(+)	(+)	(+)	(+)	(+)	(+)	(+)
Were outcome data complete without attrition or exclusion from analysis?	(+ +)	(+ +)	(+ +)	(+ +)	(+ +)	(+ +)	(+ +)	(+)	(+ +)	(+ +)	(+ +)	(+ +)	(+ +)	(+)	(+ +)	(+ +)	(+ +)
Can we be confident in the exposure characterization?	(+ +)	(+)	(+)	(+ +)	(+ +)	(+ +)	(+ +)	(+ +)	(+ +)	(+ +)	(+ +)	(+ +)	(+ +)	(+ +)	(+ +)	(+ +)	(+ +)
Can we be confident in the outcome assessment?	(+ +)	(+ +)	(+ +)	(+ +)	(+ +)	(+ +)	(+)	(+ +)	(+ +)	(+ +)	(+ +)	(+ +)	(+ +)	(+)	(+ +)	(+ +)	(+ +)
Were all measured outcomes reported?	(+)	(+ +)	(+ +)	(+ +)	(+ +)	(+ +)	(+ +)	(+ +)	(+ +)	(+)	(+ +)	(+ +)	(+ +)	(+)	(+ +)	(+ +)	(+ +)
Were there no other potential threats to internal validity (e.g., statistical methods were appropriate and researchers adhered to the study protocol)?	(+)	(+ +)	(+ +)	(+ +)	(+ +)	(+ +)	(+)	(+)	(+ +)	(+ +)	(+ +)	(+ +)	(+ +)	(+)	(+ +)	(+ +)	(+ +)

Double plus sign indicates low, single plus sign indicates probably low, single minus sign indicates probably high, double minus sign indicates high risk of bias
